# Functional development of mechanosensitive hair cells in stem cell-derived organoids parallels native vestibular hair cells

**DOI:** 10.1038/ncomms11508

**Published:** 2016-05-24

**Authors:** Xiao-Ping Liu, Karl R. Koehler, Andrew M. Mikosz, Eri Hashino, Jeffrey R. Holt

**Affiliations:** 1 Department of Otolaryngology, F.M. Kirby Neurobiology Center Boston Children's Hospital, and Harvard Medical School, Boston, Massachusetts 02115, USA; 2 Department of Otolaryngology-Head and Neck Surgery and Stark Neurosciences Research Institute, Indiana University School of Medicine, Indianapolis, Indiana 46202, USA

## Abstract

Inner ear sensory epithelia contain mechanosensitive hair cells that transmit information to the brain through innervation with bipolar neurons. Mammalian hair cells do not regenerate and are limited in number. Here we investigate the potential to generate mechanosensitive hair cells from mouse embryonic stem cells in a three-dimensional (3D) culture system. The system faithfully recapitulates mouse inner ear induction followed by self-guided development into organoids that morphologically resemble inner ear vestibular organs. We find that organoid hair cells acquire mechanosensitivity equivalent to functionally mature hair cells in postnatal mice. The organoid hair cells also progress through a similar dynamic developmental pattern of ion channel expression, reminiscent of two subtypes of native vestibular hair cells. We conclude that our 3D culture system can generate large numbers of fully functional sensory cells which could be used to investigate mechanisms of inner ear development and disease as well as regenerative mechanisms for inner ear repair.

## Abstract (web summary)

Sensory hair cells from the mammalian inner ear do not regenerate. Here, the authors induce direct hair cell formation from mouse embryonic stem cells using a three-dimensional culture system and observe differentiation of Type I and Type II vestibular hair cells and establishment of synapses with neurons.

## Graphical abstract

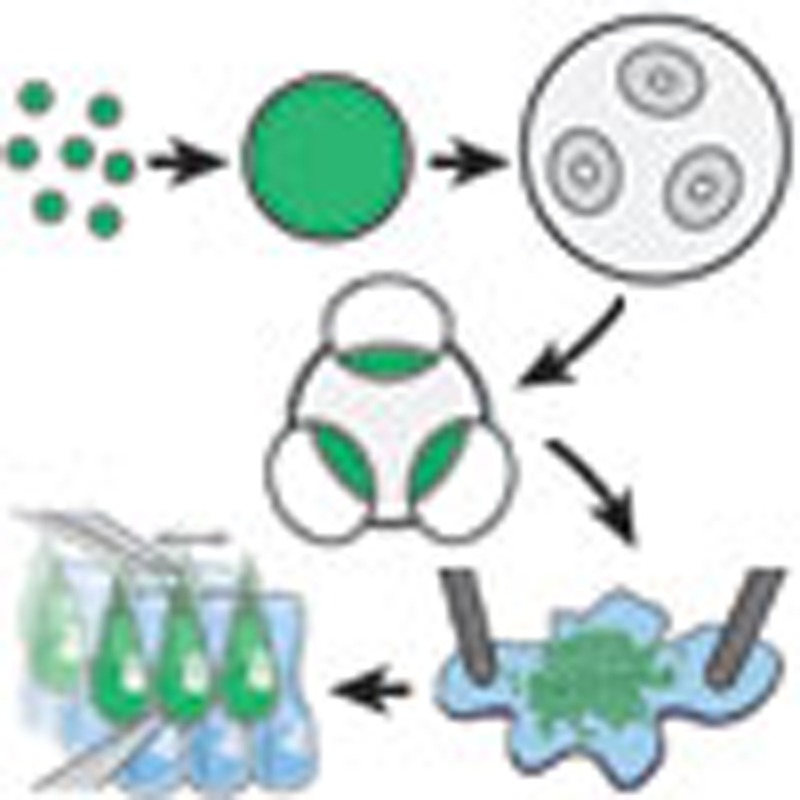

Hearing and balance rely on proper functioning of mechanosensitive hair cells in the inner ear sensory organs, consisting of the cochlea (sensitive to sound vibrations), the utricle and saccule (sensitive to head tilt and linear acceleration) and the semicircular canals (sensitive to head rotation). Hair cells transduce mechanical stimulation of their apical hair bundles into graded electrical responses that drive synaptic release onto afferent neurons. Unfortunately, hair cells are easily damaged due to acoustic overstimulation, ototoxic drugs, degeneration from genetic mutations and ageing^1^,^2^,^3^,^4^,^5^,^6^,^7^,^8^, and have limited ability to regenerate in adult mammals^9^,^10^,^11^,^12^,^13^. An *in vitro* method of producing functional hair cells could be valuable therapeutically and serve as an accessible system for studying hair cell disease, death and regeneration.

Previous attempts for generating hair cells *in vitro* used two-dimensional culture methods which resulted in low efficiency, heterogeneity and incomplete phenotypic conversion^14^. Three-dimensional (3D) culture systems have allowed researchers to generate tissues *in vitro* that resemble *in vivo* structures and organs, with potential applications to tissue engineering, drug screening, disease modelling and studies of development. We recently adapted a 3D method to create tissues that resemble inner ear sensory epithelia populated with hair cells^15^. These stem cell-derived epithelia, designated as inner ear organoids, harbour a layer of tightly packed hair cells whose structural and biochemical properties are indistinguishable from native hair cells in the mouse inner ear.

Here we assess functional properties of hair cell-like cells in inner ear organoids using single-cell electrophysiology. We find that organoid hair cells have mechanosensitivity and intrinsic electrical properties that resemble native hair cells. Interestingly, the organoid hair cells appear to develop the precise ion channel complements appropriate for particular subtypes of vestibular hair cells with distinct response properties. Vestibular hair cell ion channel expression follows a stereotyped temporal pattern during late-embryonic and early postnatal periods of development^16^,^17^, possibly in response to a cascade of precisely timed developmental signals. Organoid hair cells closely mirror this developmental pattern characteristic of *in vivo* hair cells, suggesting that the organoid microenvironment provides the proper sequential cues for normal hair cell development.

## Results

### Generation of inner ear organoids from mouse ES cells

**Figure 1:**
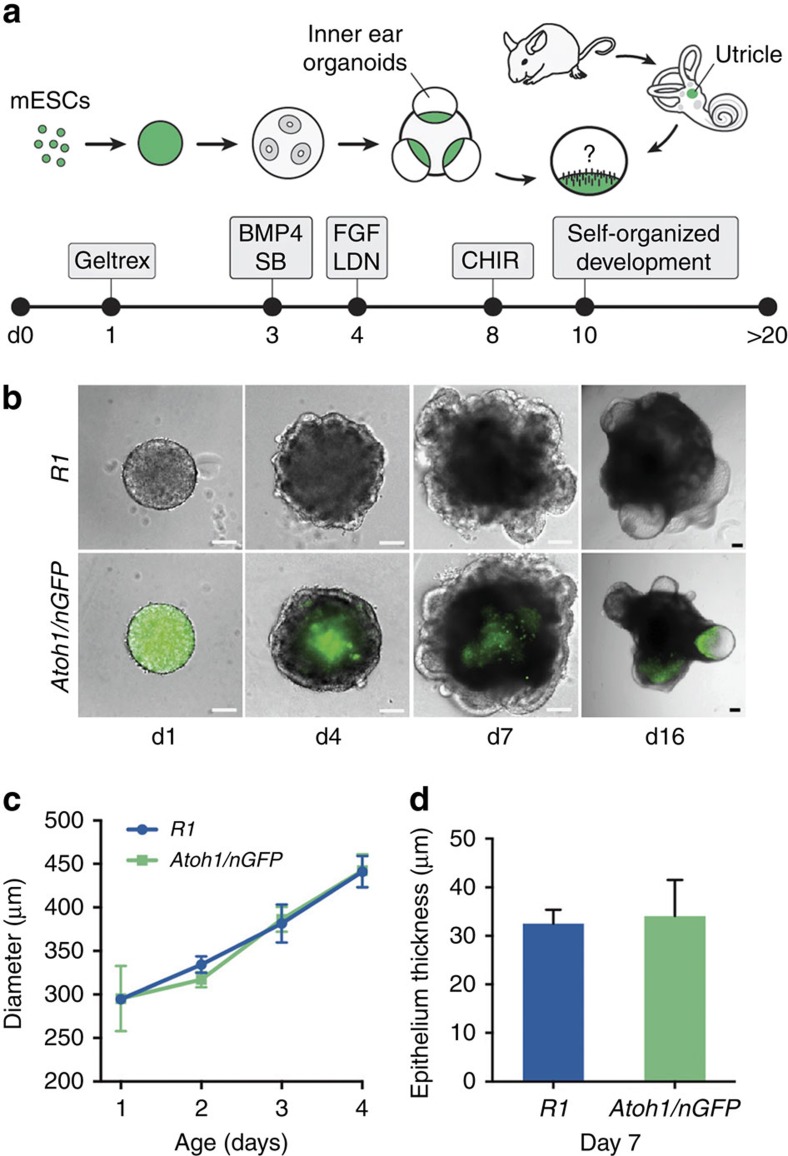
Generation of inner ear organoids from *R1* and *Atoh1/nGFP* ES cells. (**a**) Schematic overview of the inner ear induction protocol. (**b**) Comparison of aggregate morphology during otic induction in *R1* and *Atoh1/nGFP* cells. Scale bars, 100 μm. Inner ear organoids can be seen protruding from the aggregates in the day 16 panels. (**c**) The mean (±s.e.m.) diameter of *R1* and *Atoh1/nGFP* cell aggregates increases at a similar rate over time. (**d**) The mean (±s.e.m.) apparent thickness of outer epithelia on day 7 aggregates is not significantly different between *R1* and *Atoh1/nGFP* aggregates.

**Figure 2:**
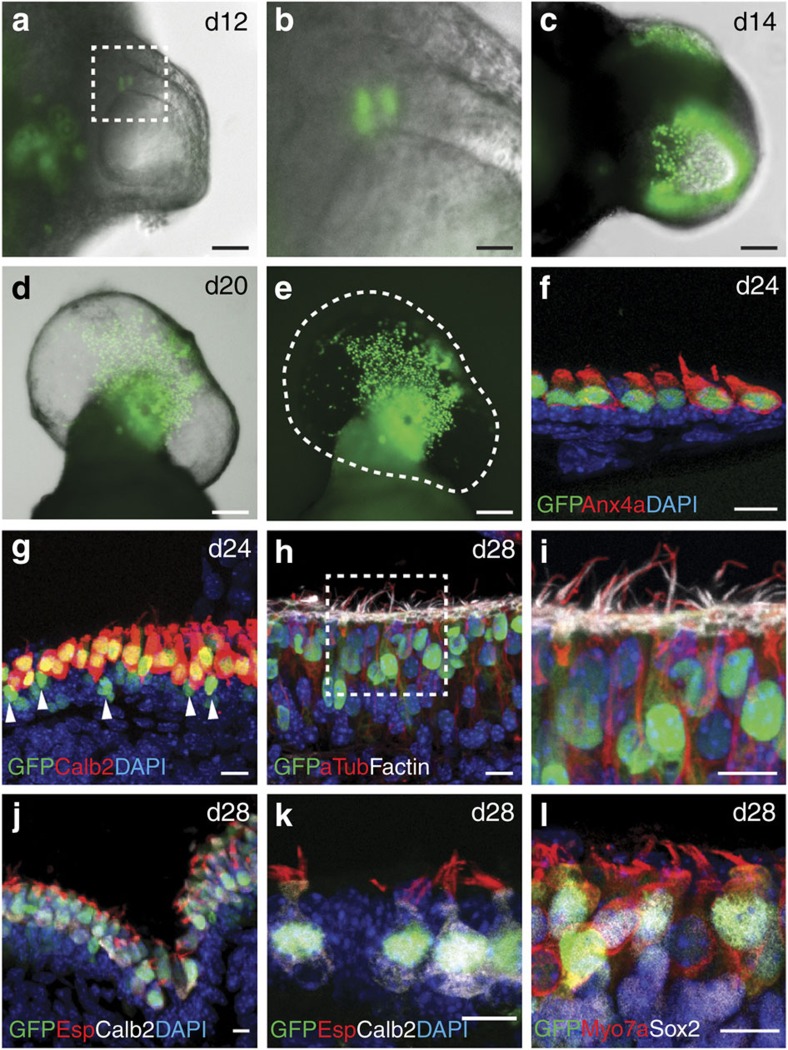
Inner ear organoids generated from *Atoh1/nGFP* ES cells include nGFP+ cells that expressed multiple hair cell markers. (**a**,**b**) The appearance of nGFP+ cells in the epithelium of organoid vesicles on day 12 of differentiation. (**c**) At later stages of differentiation (>day 14), numerous nGFP+ cells populated the epithelium, typically in a regional cluster as seen in **d** and **e**. (**f**,**g**) Most nGFP+ cells were also Anx4a+ and Calretinin (Calb2)+. Arrowheads in **g** denote nGFP+ cells devoid of Calb2. (**h**,**i**) nGFP+ hair cells had acetylated-tublin+ kinocilia and F-actin+ hair bundles. (**j**,**k**) Most Calb2+ hair cells also had Espin+ hair bundles. (**l**) nGFP+ cells were also Myo7a+ and Sox2+. Scale bars, 100 μm (**a**,**c**,**d** and **e**), 25 μm (**b**) and 10 μm (**f**–**l**).

To facilitate identification of hair cells in 3D cultures, we applied our inner ear induction protocol^15^ to *Atoh1/nGFP* mouse reporter embryonic stem (ES) cells (hereafter, *Atoh1/nGFP* cells; Fig. 1a), in which *Atoh*1+ cells expressed nuclear-localized green fluorescent protein (GFP)^14^,^15^,^18^,^19^. In the *Atoh1/nGFP* cell line, early undifferentiated cells as well as inner ear hair cells were nGFP+ (Fig. 1b). In comparison to R1 ES cells^15^, *Atoh1/nGFP* cell aggregates grew at a similar rate and generated outer epithelia that thickened following treatment with FGF2 and the BMP inhibitor LDN-193189—an indication of pre-otic induction (Fig. 1c,d). Following a pulse treatment with the Wnt agonist CHIR99021 between days 8 and 10 (D8–10), we observed inner ear organoids in 70–80% of the aggregates between D12 and 30 (Fig. 1b). The expression of GFP gradually diminished and was extinguished by differentiation day 8. Later, nGFP+ cells reemerged in organoid vesicles as early as day 12 of differentiation (Fig. 2a,b). After further development, the number of nGFP+ cells increased, forming organoid regions densely packed with nGFP+ cells (Fig. 2c–e), similar to the dense distribution of hair cells in the utricular macula. We noted that most nGFP+ cells were also immunopositive for Anxa4a, Myo7a, Calretinin(Calb2) and Sox2 with bundles immunopositive for acetylated-Tublin, F-actin and Espin (Fig. 2f–l). In three D20–24 organoids stained for hair cell markers, Myo7a, Calb2 or Sox2, we found that 68±8.6% (mean±s.e.m.) of nGFP+ were also positive for a hair cell marker. Occasionally, we observed nGFP+ cells in the supporting cell layer (Fig. 2g, arrowheads), likely indicating cells transitioning to a hair cell fate^20^. Consistent with our previous findings, the F-actin+, Espin+ hair bundles had a vestibular-like morphology (Fig. 2h–k). Together, these data demonstrate several similarities between organoid hair cells and native vestibular hair cells and indicate that *Atoh1/nGFP* reporter expression can be used to identify hair cells in ES cell-derived organoids.

### Inner ear organoids contain cells that are mechanosensitive

**Figure 3:**
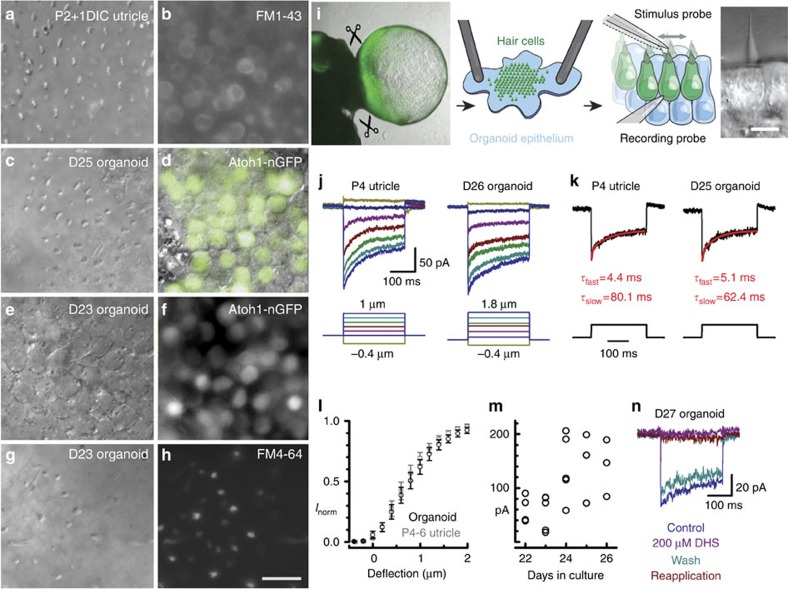
Organoid epithelia resemble sensory epithelia of native utricles and have functional mechanotransduction. (**a**–**h**) Left and right column images in the same row are paired (same tissue and region). (**a**) DIC image of a utricle epithelium from a C57BL/6J mouse at the focal plane of the hair bundles. (**b**) Fluorescence image of FM1–43 shown at the somatic plane. (**c**) DIC image of a day 25 organoid epithelium at the bundle plane. (**d**) Overlay of DIC and nGFP fluorescence images focused at the somatic level. (**e**) DIC image focused at the somatic level. (**f**) Fluorescent image of the same field shown in **e** showing Atoh1-nGFP+ cell bodies. (**g**,**h**) DIC and FM4–64 fluorescence images focused at the hair bundle level of the same D23 organoid epithelium shown in **e** and **f**. Scale bar, 20 μm applies to panels **a**–**h**. (**i**) Schematic of preparation for organoid electrophysiology. Organoids were dissected (left), and the epithelium was flattened and pinned with hair bundles facing up (middle left). Single hair bundles (right) were mechanically stimulated during whole-cell recording from the cell body (middle right). Scale bar, 10 μm. (**j**) Families of mechanotransduction currents evoked by step bundle deflection protocols shown below. (**k**) Single transduction current traces measured at the half-maximal deflection (0.4 μm left, 0.8 μm right), fitted with double exponential curves (red). (**l**) Mean±s.e.m. current-displacement relations for 10 organoid and 8 utricle hair cells. (**m**) Transduction current amplitudes for organoid hair cells plotted as function of days in culture. (**n**) Transduction currents before, during, after and following reapplication of the transduction channel blocker 200 μM dihydrostreptomycin (DHS).

Further inspection of well-developed *Atoh1/nGFP* organoid epithelia revealed many similarities to native utricle sensory epithelia. In utricles, planar polarity of bundle orientation is highly organized (Fig. 3a) and nuclei lie in a focal plane below the bundles (Fig. 3b). In the ESC-derived sensory epithelia, hair bundles often showed local alignment (Fig. 3c) with nGFP+ nuclei visible in deeper focal planes (Fig. 3d). Hair cells that have functional mechanotransduction can be labelled with brief application of the vital dye, FM1–43, which enters through open transduction channels (Fig. 3b). Due to spectral overlap between nGFP and FM1–43, we used an alternative dye, FM4–64, which also enters large cation channels and has excitation and emission spectra shifted to longer wavelengths. To assay for functional mechanosensitive channels in ESC-derived organoids, we applied FM4–64 and imaged at the bundle level. nGFP+ nuclei were visible at the cell body level (Fig. 3e,f) and FM4–64 fluorescence was evident in cell bodies and hair bundles of nGFP+ cells (Fig. 3g,h), suggesting the presence of functional mechanotransduction channels. While FM dyes are useful for visualization of large numbers of cells, measurements from individual cells are required to definitively identify mechanosensitive hair cells.

To more precisely assess the functionality of these ESC-derived hair cells and their similarity to native hair cells, we performed electrophysiological recordings using the whole-cell, tight-seal technique (Fig. 3i–n). The following electrophysiology data are taken from 153 cells in ESC-derived sensory epithelia, 10 derived from R1 embryonic stem cells and 143 from *Atoh1/nGFP* stem cells.

Hair cell mechanosensitivity depends on ion channels, located in the hair bundle, that open in response to bundle deflection towards the tallest stereocilia^21^,^22^. We examined 62 cells with intact bundles and well-coupled stimulus probes. Forty-three of the 62 cells (69%) were mechanosensitive (Fig. 3j–n), with amplitudes (Fig. 3m), adaptation (Fig. 3j,k) and displacement sensitivity (Fig. 3l) similar to native vestibular hair cells. Since the properties of mechanotransduction vary by hair cell type and region^23^, we performed a quantitative comparison of organoid hair cells relative to utricle extrastriolar hair cells that had morphological and electrophysiological properties typical of Type II cells (Table 1). We found that mechanotransduction current amplitudes and time constants of adaptation in organoid hair cells were consistent with those of the P4 utricle hair cells in our sample and those reported previously^24^,^25^,^26^,^27^. The time constants for organoid hair cells (<10 ms for *τ*_fast_ and tens of ms for *τ*_slow_) were not significantly different than those of utricle hair cells (*P*=0.6 and 0.09 *t*-test, respectively; Table 1). In contrast, adaptation in cochlea hair cells is an order of magnitude faster (<1 ms for *τ*_fast_ and <10 ms for *τ*_slow_ (refs 28, 29, 30, 31, 32)). The 10–90% operating range was also larger (*P*=0.03) and extent of adaptation smaller for ESC-derived hair cells (*P*=0.02; Table 1) than for utricle hair cells. The average maximal transduction amplitude was 99±12 pA (*n*=30), smaller than typically seen in postnatal utricle (150–250 pA). However, the organoid transduction current amplitudes appeared to increase between D23 and D25 of culture, and attained amplitudes similar to native hair cells by D25 (Fig. 3m, Table 1; *P*=0.77), but at a slower rate. The onset of mechanotransduction in native vestibular hair cells is rapid, going from zero to mature current amplitudes over the course of one day^16^. We confirmed that these stimulus-evoked currents in organoid hair cells could be reversibly blocked by the transduction channel blocker dihydrostreptomycin (Fig. 3n), consistent with the pharmacology of native hair cells^33^.

### Organoid hair cells express voltage-dependent currents

We also examined other membrane currents seen in native utricle hair cells. Ion channels present on the hair cell basolateral membrane are critical for normal hair cell function and shape the voltage signal that determines the synaptic output of the cells. Voltage-dependent currents in ESC-derived hair cells resembled vestibular hair cells and followed temporal patterns of ion channel expression seen in developing vestibular hair cells. In cells with morphology resembling hair cells, we observed negative resting potentials, the presence of large outward K^+^ currents, and fast inward rectifying K^+^ currents. The majority of these cells had membrane currents resembling Type II vestibular hair cells, but we observed six cells in five different organoids that resembled Type I vestibular hair cells. Additionally, in some nGFP+ cells that lacked hair bundles, we observed small outward K^+^ currents (771±121 pA at +35 mV; *n*=7), but no fast inward rectifying K^+^ currents. These cells may represent very immature hair cells as the outward K^+^ current amplitudes were similar to E15, but the lack of inward rectifier and hair bundles suggested they were more similar to E14 vestibular cells^17^. Alternatively they may have been destined for another cell fate and thus were excluded from subsequent analysis. Capacitance of hair cell-like cells (including Type I's, D22–28) averaged 6.0±0.2 pF (*n*=136), slightly higher but in the same range of cell capacitance reported for neonatal utricle hair cells (Table 1; *P*=0.07).

**Figure 4:**
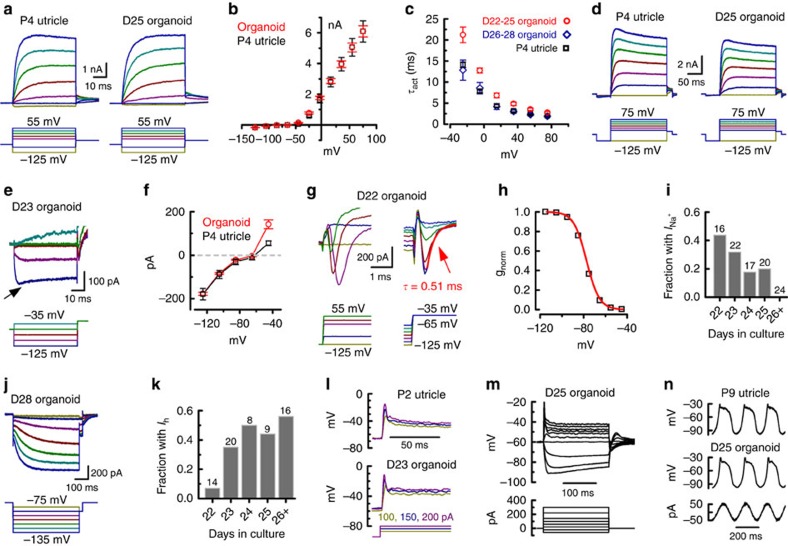
Electrophysiological properties of organoid hair cells. (**a**) Families of outward K^+^ currents evoked by the voltage protocol shown below. (**b**) Mean±s.e.m. peak current–voltage relations for 27 organoid hair cells (D22–28) and eight Type II utricle hair cells (P4). (**c**) Mean±s.e.m. time constants of single exponential fits to K^+^ current activation as function of membrane potential for D22–25 (*n*=12), D26–28 (*n*=6) organoid hair cells and P4 utricle hair cells (*n*=7). (**d**) Families of K^+^ current evoked by a voltage protocol (below) with a prepulse to −125 mV. (**e**) A family of fast inward rectifier currents recorded from a D23 organoid hair cell evoked by the protocol shown below. (**f**) Mean±s.e.m. peak inward rectifier current–voltage relations for 27 organoid hair cells and eight utricle hair cells. (**g**) Families of Na^+^ current activation (left) and inactivation (right). (**h**) The voltage dependence of Na^+^ current inactivation extracted from the peak inward currents (panel **g**, right) plotted as a function of prepulse potential and fit by a Boltzmann curve with *V*_1/2inact_=−78.3 mV and *s*=5.9 mV. (**i**) Prevalence of Na^+^ current in organoid hair cells as a function of days in culture. Total number of cells for each age is indicated above the bars. (**j**) A family of slow hyperpolarization-activated currents (*I*_h_) evoked by the protocol below. (**k**) Prevalence of *I*_h_ in organoid hair cells as function of days in culture. Number of cells is indicated above each bar. (**l**) Representative membrane responses of utricle and organoid hair cells to step current injections (below). (**m**) A family of voltage responses evoked by incremental current steps. (**n**) Representative utricle and organoid hair cell membrane responses to sinusoidal current injections.

All Type II-like ESC-derived hair cells had at least two potassium current components, a large delayed rectifier (Fig. 4a), and a smaller inward rectifier (Fig. 4e, arrowhead), both characteristic of Type II utricle hair cells. The delayed rectifier current was several nanoamps in size, consistent with that observed during the first postnatal week in the utricle^17^,^34^,^35^. The delayed rectifier current dominated the current–voltage relationships, similar to those of native utricle hair cells (Fig. 4b). Delayed rectifier K^+^ current size increased with number of days in culture (at nominal +95 mV, D22–23: 5.7±0.3 nA, *n*=25; D24–25: 7.5±0.4 nA, *n*=16; D26–28: 8±0.5 nA, *n*=17) consistent with K^+^ current development in perinatal utricle hair cells^17^,^34^,^35^. The maximal chord conductance approximated by fitting the linear region of the averaged *I*–*V* curve (*n*=27) was 54 nS. The activation time course for the delayed rectifier current was slower in D22–25 ESC-derived hair cells than for P4 utricle recordings (Fig. 4c) and slower than reports in the literature^17^,^34^,^35^. Between D26 and D28, activation in 6/12 cells were very poorly fit by a single time constant, but well fit by the sum of two exponentials. In the remaining six cells that were reasonably fit, the time constants were similar to values obtained in utricle hair cells (Fig. 4c).

The delayed rectifier showed minimal inactivation (<2% of peak) during 50 ms voltage steps and was relatively uniform across the population of ESC-derived cells, which contrasts with the presence of subpopulations of hair cell-like cells with rapidly inactivating outward current in Oshima *et al*.^14^. In response to another protocol with a longer depolarizing step and a hyperpolarizing prepulse, ESC-derived hair cells also exhibited similar voltage-dependent outward current properties as utricle hair cells (Fig. 4d).

In response to hyperpolarization to −125 mV, a rapidly activating inward current was evoked (Fig. 4e) that can also be seen in the *I*–*V* curves as an inwardly rectifying region in the hyperpolarized range (Fig. 4f). This current resembled *I*_K1_, a fast inward rectifier K^+^ current, prominent in vestibular hair cells^17^,^34^,^36^,^37^. Notably, Levin and Holt^38^ found an absence of inward rectifier current in vestibular hair cells of Kir2.1-deficient mice. Comparison of current-clamp recordings from Kir2.1-deficient and wild-type mice suggested the current is active at rest, has a hyperpolarizing effect on the resting membrane potential, and speeds up membrane responses from rest. An inward rectifier current also appears transiently in developing cochlear hair cells^39^. The near maximal conductance of *I*_K1_, calculated between −105 and −125 mV from the average *I*–*V* curves was 4.5 nS for P4 utricle (*n*=8) and 4.7 nS for ESC-derived hair cells (*n*=27).

In addition to K^+^ currents, we also observed other currents typical of vestibular hair cells. Although mature hair cells do not fire action potentials, small Na^+^ currents are transiently expressed in developing hair cells and may play a developmental role^17^,^40^. The currents consist of tetrodotoxin (TTX)-sensitive, TTX-insensitive Na_V_1.5-like, or both, depending on hair cell type, anatomical zone and developmental age^23^. In many ESC-derived hair cells, we observed inward Na^+^ currents with rapid voltage-dependent activation and inactivation (Fig. 4g,h). Utricle hair cell Na^+^ currents peak in amplitude between E16 and P1 and then decline into adulthood^17^,^23^,^40^. Remarkably, we observed the same pattern of declining prevalence as the ESC-derived hair cells matured (Fig. 4i).

Vestibular hair cells, but not cochlear hair cells express *I*_h_, a hyperpolarization-activated mixed cation current flowing through channels whose subunits are encoded by the *HCN1*–*4* genes^34^,^36^. In vestibular hair cells, HCN1 is the main contributor to *I*_h_ based on quantitative RT–PCR (PCR with reverse transcription) and recordings from *Hcn1*^−/−^, *Hcn2*^−/−^ and *Hcn1/2*^−/−^ mice^41^. *I*_h_ can be identified as an inward current that activates very slowly upon hyperpolarization (Fig. 4j). *I*_h_ was evident in 25/67 ESC-derived hair cells. *I*_h_ increases greatly in prevalence and size between P3 and P8 (refs 34, 41), and was not observed by Géléoc *et al*.^17^ who recorded through P2. Likewise, the prevalence of I_h_ in ESC-derived hair cells increased with number of days in culture (Fig. 4k). At D28, *I*_h_ was prominent and a depolarizing ‘sag' characteristic of cells with *I*_h_ could be seen in response to large hyperpolarizations (Supplementary Fig. 1B). In D27–28 cells, the major time-dependent component of *I*_h_ activation at −125 mV was well fit by a single exponential with *τ*=181±26 ms (*n*=7), slower than for P8–P10 hair cells^41^, but still within the range reported for HCN1 and faster than for the other HCN subunits^42^. Boltzmann fits of the voltage-dependent activation of *I*_h_ had similar *V*_1/2_ and slope values as utricle hair cells (Supplementary Fig. 1A).

### Organoid hair cells have normal voltage responses

To test the membrane responses of the ESC-derived hair cells, we performed recordings in current-clamp mode. The mean resting potential of ESC-derived hair cells was −63.2±0.7 mV (*n*=38), similar to P4 utricle hair cells (Table 1). Step current injections in ESC-derived Type II-like hair cells produced relatively large voltage excursions (Fig. 4l,m). In both cases, membrane potential peaked near −20 mV and then repolarized as outward K^+^ currents activated. The steady-state voltage in the hyperpolarizing direction was larger than in the depolarizing direction (Fig. 4m), due to small inward rectifier K^+^ current and large outward rectifying K^+^ currents, respectively. The membrane voltage followed sinusoidal currents (mimicking oscillating stimuli like walking, vibration, or acoustic stimuli) and was also characterized by an initial peak and larger deflections in the hyperpolarizing direction (Fig. 4n, ∼5 Hz). A frequency modulated sweep protocol spanning 1–30 Hz was sometimes applied; the organoid hair cells all displayed high pass properties, presumably due to activation of K^+^ currents at lower frequencies (Supplementary Fig. 1C). The responses were similar to those of P4 vestibular hair cells (Fig. 4l,m) and to vestibular cells described previously^38^,^43^.

### Inner ear organoids have features of functional synapses

**Figure 5:**
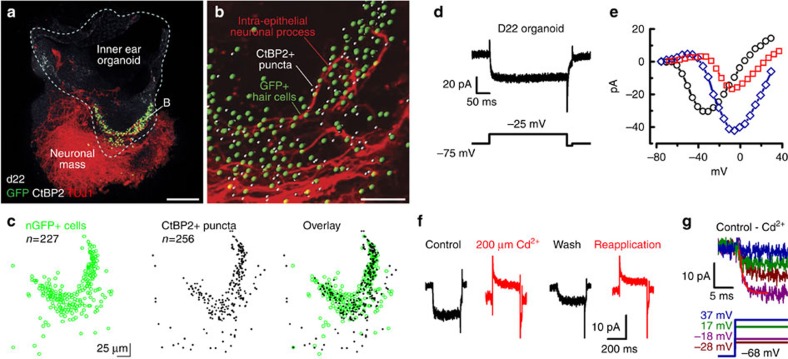
*Atoh1/nGFP* organoid hair cells have features that typify vestibular hair cell synapses. (**a**,**b**) Confocal image of nGFP+ organoid hair cells with CtBP2+ puncta that colocalize with TUJ1+ neuronal processes. Scale bars, 50 μm (**a**), 20 μm (**b**). (**c**) Quantitative colocalization analysis of segmented CtBP2+ punta and nGFP+ nuclei. (**d**) A non-inactivating Ca^2+^ inward current evoked by the voltage step shown below. (**e**) Ca^2+^ current–voltage relations from three organoid hair cells. (**f**) Ca^2+^ currents evoked by a step to −23 mV were blocked by 200 μM Cd^2+^. (**g**) A family of Ca^2+^ currents evoked by the voltage protocol shown below. Subtraction of traces recorded in 200 μM Cd^2+^ from control traces revealed the Ca^2+^ currents shown. Activation kinetics were fit with a single exponential with a time constant of 1.1 ms at −25 mV (red line).

In day 20–30 organoids, we observed synapse-like CtBP2+ puncta associated with TUJ1+ neural processes at the base of nGFP+ cells (Fig. 5a,b; Supplementary Fig. 2; Supplementary Movies 1 and 2). To facilitate visualization, we plotted the spatial coordinates of each nGFP+ cell and each CtBP2+ punctum and found that 87% (223/256) of the puncta were closely associated (<5 μm) with nGFP+ cells (Fig. 5c; Supplementary Fig. 2), suggesting the presence of presynaptic release sites in organoid hair cells.

Neurotransmitter release in hair cells is dependent on calcium entry through voltage-gated Ca^2+^ channels, so we sought to determine whether Ca^2+^ currents were present in ESC-derived hair cells. Hair cells have a non-inactivating or weakly inactivating L-type Ca^2+^ current; cochlear inner hair cells differ from vestibular hair cells in having a much larger Ca^2+^ current that is almost entirely dependent on Ca_V_1.3 (refs 44, 45, 46, 47, 48, 49). In inner hair cells, this current also participates in calcium-based action potentials during development^50^,^51^,^52^. We isolated Ca^2+^ currents with an external solution containing 140 mM TEA, 10 mM 4-AP and 5 mM Ca^2+^. In 12/17 cells, we observed inward currents that activated with depolarization and displayed very little or no inactivation (Fig. 5d). These currents were typically small (15–35 pA), but within the range previously described for vestibular hair cells^44^,^46^,^47^,^48^. The peak inward currents were evoked by steps to between −40 and 0 mV (Fig. 5e), as previously described for vestibular hair cells^44^,^46^,^48^. The current was blocked by a Ca^2+^ channel blocker, Cd^2+^ (Fig. 5f); subtraction of the currents in Cd^2+^ revealed an inward current with an activation time course similar to that reported previously for vestibular hair cell Ca^2+^ currents (Fig. 5g)^44^. Therefore, organoid hair cells have calcium currents that resemble those of vestibular hair cells and may be sufficient for synaptic release onto TUJ1+ neurites (Fig. 5a,b; Supplementary Fig. 2; Supplementary Movies 1 and 2). Occasionally (*n*=4), we encountered nGFP-negative cells that had currents resembling those typical of vestibular ganglion neurons (Supplementary Fig. 3A). The cells had large rapidly activating and rapidly inactivating inward currents (Supplementary Fig. 3B–D) with properties similar to those of sodium currents previously described in vestibular ganglion neurons^53^,^54^.

### Some organoid hair cells have features of Type I hair cell

**Figure 6:**
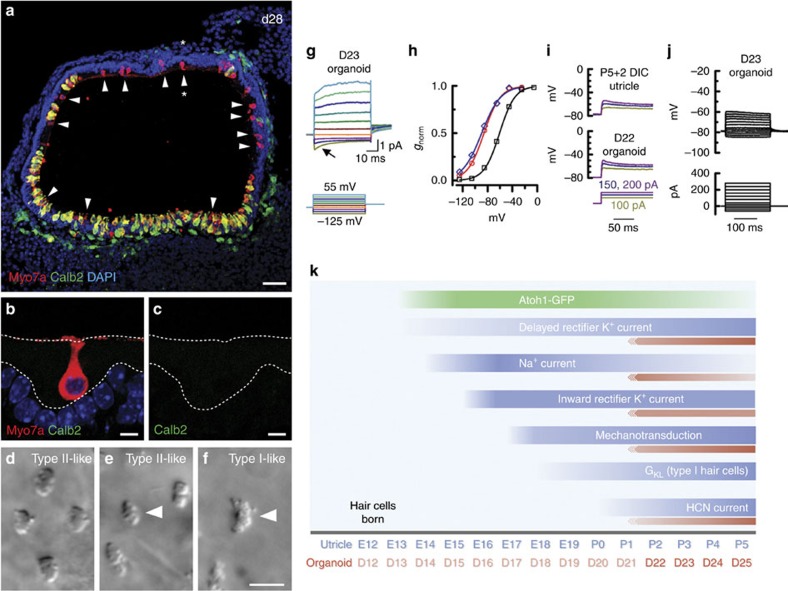
Evidence of Type I hair cells in ES cell-derived organoids. (**a**) Confocal image of a day 28 organoid reveals numerous Myo7a+, Calb2- hair cells (arrowheads). (**b**,**c**) High magnification image of a Myo7a+, Calb2- Type I-like hair cell in **a** (asterisk). (**d**–**f**) DIC images of organoid hair bundles with Type II- (**d**,**e**) and Type I- (**f**) like morphology. Type II- (**e**) and Type I- (**f**) like voltage-dependent currents were recorded from the cells at the centre of these images (arrows). (**g**) Family of low-voltage-activated K^+^ currents, typical of vestibular Type I hair cells. The current is active at rest, deactivates with hyperpolarization (arrow) and activates further with depolarization. (**h**) Instantaneous tail currents at −35 mV plotted as a function of prepulse voltage for three Type I-like organoid hair cells. The data were fit with Boltzmann functions where *V*_1/2_=−90, −61 and −86 mV. (**i**) Membrane responses of a utricle Type I hair cell and a Type I-like organoid hair cell evoked by the current steps shown below. (**j**) Family of membrane responses from a Type I-like organoid hair cell evoked by the current injection protocol shown (compared with Fig. 4m). (**k**) Summary time line of the functional maturation of mechanotransduction and ion channel acquisition for utricle hair cells (blue) and organoid hair cells over time in culture (red). Scale bars: 25 μm (**a**), 10 μm (**d**,**e** and **f**) and 5 μm (**b**,**c**).

In the vestibular system, there are two types of hair cells with different protein expression profiles, cellular and bundle morphology, electrophysiological properties, synaptic connectivity and zonal distribution. Calbindin-2 (Calb2) expression is known to distinguish Type II (Calb2+) from Type I (Calb2−) hair cells. In D23–28 organoids, the majority of Myo7a+ hair cells were Calb2+; however, a subset of hair cells was Calb2−, suggesting a Type I identity (Fig. 6a–c) and several cells had broader hair bundles typical of Type I cells. While the majority of organoid hair cells had hair bundles (Fig. 6d,e) and electrophysiological phenotypes typical of Type II vestibular hair cells, we recorded from six cells with hair bundles (Fig. 6f) and morphologies that resembled Type I hair cells. Type I hair cells can be identified electrophysiologically by the presence of a large outward rectifier K^+^ conductance with a negative activation range referred to as *G*_K,L_ (refs 55, 56, 57, 58), which is evident as early as E18 (ref. 17). *G*_K,L_ becomes prominent between P4 and P8 and is partially active at the resting potential^34^, contributing to negative resting potentials, low input resistances, linearization of voltage responses to incremental current stimulation and fast membrane time constants. Based on voltage dependence, kinetics and pharmacology, *G*_K,L_ may be composed of different ion channels, with KCNQ-like currents at immature stages^59^ and an ether-a-go-go (erg)-like current at more mature stages^35^. In the six Type I-like cells, voltage steps resulted in instantaneous current changes due to *G*_K,L_ active at rest (Fig. 6g). A step to −125 mV produced an inward K^+^ current that deactivated with a primary time constant of 9.8±0.8 ms (*n*=6) in accord with Rüsch and Eatock^58^. Tail currents taken at −35 mV revealed K^+^ current that activated very negatively (Fig. 6h), possibly with two distinct contributors with half activation around −60 and −80 mV, as previously described for *G*_K,L_ (refs 35, 58). Consequently, the resting potentials were 10–20 mV more negative and input resistances more than an order of magnitude lower than for the Type II-like ESC-derived hair cells, and in close agreement with utricle Type I hair cells (Table 1). Membrane time constants, measured in current-clamp mode, were more than tenfold faster for Type I-like hair cells (0.52 and 0.97 ms) than Type II-like hair cells (15±1.9 ms, *n*=19), consistent with the more than tenfold decrease in input resistance. Current-clamp recordings showed Type I-like cells (Fig. 6i) had smaller voltage responses to current injections than Type II-like cells (Fig. 4l), consistent with a significant K^+^ conductance active at rest. Type I-like cells also had a more linear input–output relationship for current injection (compare Figs 6j and 4m), as previously shown for cells with *G*_K,L_. The speed and linearity of responses is well-suited for vestibular compensatory reflexes which show near unity gain and minimal phase lag across a variety of stimulus parameters^60^,^61^,^62^,^63^.

## Discussion

Stem cell-based therapeutics have great potential for treating disorders caused by cell loss. Partial successes in treating retinal disease^64^,^65^ and Parkinson's disease^66^ with transplantation of stem cell-derived cells form a proof-of-concept for the efficacy and safety of the approach. Replacement of lost sensory hair cells may be a viable treatment strategy for hearing and balance disorders. However, because these cells exhibit great diversity in their functional properties, which depend largely on their ion channel complement, generation of suitable replacement cells must precisely recreate the properties of native sensory cells. Previously, Koehler *et al*.^15^ established a novel method for generating hair cell-like cells from mouse ESCs. Rather than directly stimulating various pathways in a monolayer, ESCs were guided toward a placodal fate and then allowed to develop in 3D culture into organoids in a self-organizing process. Here we show that hair cells created with this method progressed with a temporal pattern of ion channel expression that closely resembled either of two types of native vestibular hair cells.

In developing vestibular organs, expression of the basic-helix-loop-helix transcription factor Atoh1/Math1 begins at E12.5 (refs 67, 68) and is required for terminal differentiation of sensory hair cells. In *Atoh1/nGFP* mice, nGFP is detectable in developing hair cells beginning around E13.5 (ref. 19). Remarkably, Atoh1-GFP expression followed a similar time course in developing organoids, with Atoh1-nGFP signal appearing as early as D12. If we take the first appearance of Atoh1-nGFP in native vestibular organs and in ESC-derived organoids as equivalent stages, then D22 through D25 in the organoids corresponds to approximately the P2–P5 period in mouse development, given the mouse gestation period of ∼20 days.

The development of organoid hair cells showed developmental patterns reminiscent of native vestibular hair cells, although slightly delayed. In the vestibular sensory epithelium, outward rectifying K^+^ currents appear around E14 and increase in amplitude over the subsequent days, followed by the appearance of fast inward rectifier currents at E15; Na^+^ currents peak at E16 and then decline; transduction appears around E16; and *G*_K,L_ (in Type I hair cells) and HCN currents gradually become more prominent over the first postnatal week (refs 17, 34, 41); Fig. 6k). The temporal pattern of ESC-derived hair cell progression between D22 and D25 was consistent with the sequence of native vestibular hair cell development: we measured increasing K^+^ current amplitudes, presence of inward rectifier currents throughout, decreasing Na^+^ currents, increasing HCN currents, occasional *G*_K,L_, and functional mechanotransduction. That the developmental sequence in organoid hair cells was similar to that of native utricle hair cells lends confidence that our ESC-derived cells are bona fide hair cells. The reason for the developmental delay in the organoids is unclear, though we note differences in mouse strains may provide an explanation. Alternatively, we speculate that the bath culture system in which the organoids were maintained may have diluted secreted signalling molecules that would otherwise be more concentrated in the confined spaces of the developing inner ear.

By D25, we found that hair cells from inner ear organoids had fully functional mechanotransduction and voltage-gated currents, including calcium currents required for synaptic release. The inner ear contains multiple types of hair cells including cochlear inner and outer hair cells and vestibular Type I and Type II hair cells. The organoids morphologically resembled sensory epithelia of the vestibular end organs, and their hair cells stained for Calb2 (ref. 15), which in mature vestibular organs is confined to Type II hair cells^57^. However, Calb2 expression changes during development and is initially present in all hair cells^69^. We found, based on electrophysiology, most of the ESC-derived hair cells resembled vestibular Type II hair cells. The complement of voltage-gated currents in organoid hair cells included currents known to be expressed in Type II hair cells, and were more similar to those than to other types of hair cells. Voltage-gated K^+^ currents were fairly consistent across the population of our ESC-derived hair cells in contrast to Oshima *et al*.^14^ who observed greater heterogeneity in their stem cell-derived hair cells. They hypothesized that the heterogeneity was due to lack of appropriate signalling required to promote maturation into specific hair cell subtypes. Here we demonstrate that organoid hair cells were capable of differentiating and maturing into at least two hair cell subtypes. We recorded from six cells with an electrophysiological phenotype typical of Type I hair cells; all six cells expressed a large low-voltage-activated potassium conductance that confers functional properties distinct from those of Type II hair cells. Type I-like organoid cells resembled utricle Type I hair cells in their resting membrane potential, excitability, and speed of response. Correspondingly, immunohistochemistry at D28 showed a population of hair cells that were Myo7a+ but devoid of Calb2 and that had prominent ‘necks' in their morphology, providing further similarities to vestibular Type I hair cells.

Relative to the appearance of Type II hair cells, Type I hair cells become distinct later in development^34^ and during regeneration^70^. The low prevalence of Type I hair cells in organoids may simply reflect fewer days in culture. Alternatively, cochlear hair cells and Type I hair cells appeared later in evolution and expansion of their numbers may require additional signalling factors. Nevertheless, it is possible that differentiating hair cells transplanted into an existing cochlear or vestibular epithelium would further refine their properties following local signals from their immediate environment.

The amplitude, time course and sensitivity of mechanotransduction in organoid hair cells were similar to those of vestibular but not cochlear hair cells. In the utricle, the developmental acquisition of mechanotransduction occurs rapidly, proceeding from no mechanosensitivty at E15 to near mature amplitudes by E17 (ref. 16). In the organoids, we saw a more gradual rise in transduction current amplitudes. Operating range, time constants of adaptation and extent of adaptation also seemed to mature more gradually in ESC-derived hair cells than in native utricle hair cells. While the absolute values of these mechanotransduction parameters at later stages were similar to those of utricle hair cells, the temporal pattern in the organoid hair cells was more similar to the gradual maturation of mechanotransduction in cochlear hair cells^28^.

One possible explanation is that the mean values for the organoids were from pooled hair cell data acquired from cells that may have been ‘born' at different time points during organoid growth, which may have led to temporal smearing of the data. In the utricle, mechanotransduction onset appears to be tightly synchronized among cells, perhaps due to an endogenous signal. Another possibility for the extended development of bundle structure and mechanotransduction in organoid hair cells is the lack of a well formed otolithic membrane and otoconia, which are present at embryonic stages in developing utricles. Perhaps the structures serve to provide an appropriate stimulus and correct developmental cues that synchronize hair cell development *in vivo*.

Our findings suggest that the journey of normal development may be important for arrival at a particular phenotypic destination. The temporal pattern of sequential and transient ion channel expression that we observed in developing organoids may be incidental or may play a key role in development itself. Our results also suggest that many aspects of native hair cell development and cellular phenotype are normal in these organoids, validating their usefulness for the *in vitro* study of development and disease modelling. For instance, with genome editing tools, such as CRISPR/Cas9, inner ear organoids could provide a useful model to assay the role of genes and proteins involved in hair cell development, structure, function and dysfunction without the expense and time needed to generate transgenic mouse lines. The method could also be expanded to yield large numbers of ESC-derived hair cells *in vitro*, which may facilitate biochemical and high-throughput screens that are typically limited by the paucity of hair cells in native inner ear tissue. Lastly, we have not overlooked the fact that our ESC-derived organoids closely resemble native vestibular organs and may provide a source of replacement hair cells or possibly replacement of entire sensory end organs for patients who suffer vestibular dysfunction.

## Methods

### ES cell culture

ES cells were maintained in feeder-free conditions using 2i-LIF medium^18^. The R1 and *Atoh1/nGFP* (Tg(Atoh1-GFP)1Jejo) were a gift from Stefan Heller^14^,^19^. ES cells were maintained under identical conditions. Briefly, ES cells were grown on gelatin-coated plates in N2B27 medium consisted of a 1:1 mixture of Advanced DMEM/F12 and neurobasal medium (Invitrogen) supplemented with 1 mM GlutaMax (Invitrogen) and 1 mM penicillin/streptomycin (STEMCELL Technologies). 2i-LIF medium was made by supplementing N2B27 medium with 1,000 U ml^−1^ leukemia inhibitory factor (LIF; Millipore), 3 μM CHIR99021 (Stemgent) and 1 μM PD0325901 (Santa Cruz).

ES cell differentiation was performed as described previously, with slight modifications^18^. Briefly, ES cells were dissociated with 0.25% Trypsin-EDTA, resuspended in differentiation medium and plated 100 μl per well (3,000 cells) on 96-well low-cell-adhesion U-bottom plates (Lipidure Coat, NOF). Differentiation medium was G-MEM supplemented with 1.5% knockout serum replacement (KSR; Invitrogen), 0.1 mM non-essential amino acids, 1 mM sodium pyruvate, 1 mM penicillin/streptomycin and 1 mM 2-mercaptoethanol. On day 1, half of the medium in each well was exchanged for fresh differentiation medium containing Matrigel or Geltrex (2% v/v final concentration). On day 3 of the protocol, BMP4 (10 ng ml^−1^) and SB-431542 (1 μM) were added to each well at 5 × concentration in 25 μl of fresh media. On day 4–5, FGF2 (25 ng ml^−1^) and LDN-193189 (100 nM) were added to each well at 6 × concentration in 25 μl of fresh media. The concentration of Matrigel was maintained at 2% (v/v) throughout days 1–8. On day 8 of differentiation, cell aggregates were washed twice with PBS before being transferred to 96-well plates (Lipidure Coat, NOF) in N2 medium containing 1% Matrigel (v/v) and 3 μM CHIR99021 (Stemgent). N2 Medium contained Advanced DMEM/F12, 1X N2 Supplement, 1 mM penicillin/streptomycin or 50 μg ml^−1^ Normocin (Invivogen) and 1 mM GlutaMax. After 48 h the cell aggregates were transferred to 24-well plates (Lipidure Coat, NOF; 1–2 aggregates per well) in N2 medium. Half of the medium was changed every other day during long-term floating culture for up to 30 days.

### Signalling molecules and recombinant proteins

The following small molecules and recombinant proteins were used: recombinant human BMP4 (10 ng ml^−1^; Stemgent), human FGF2 (25 ng ml^−1^; Peprotech), SB-431542 (1 μM; Tocris Bioscience), and LDN-193189 (100 nM; Stemgent). Notably, we have obtained comparable results using concentrations of up to 1 μM LDN-193189.

### Immunohistochemistry

Aggregates were fixed with 4% paraformaldehyde for 20–30 min. The fixed specimens were cryoprotected with a graded treatment of 10, 20 and 30% sucrose and then embedded in tissue freezing medium. Frozen tissue blocks were sectioned into 10 or 12 μm cyrosections. For immunostaining, a 3% Goat or Horse Serum and 0.1% Triton-X100 solution was used for primary antibody incubation. An Alexa Fluor 488, 568 or 647 conjugated anti-mouse IgG or anti-goat IgG and an Alexa Fluor 568 or 647 conjugated anti-rabbit IgG (Invitrogen) were used as secondary antibodies. A DAPI counterstain was used to visualize cellular nuclei (ProLong Gold antifade reagent with DAPI, Life Technologies). Microscopy was performed on a Nikon TE2000 Inverted Microscope or an Olympus FV1000-MPE Confocal/Multiphoton Microscope.

Wholemount immunostaining was performed using the Scale clearing method as previously described^14^ 3D volume rendering and segmentation was performed by loading Olympus oif image files in Imaris 8 software (Bitplane) at the Indiana Center for Biological Microscopy. For the segmentation analysis in Fig. 5a–c and Supplementary Movie 1, nGFP+ nuclei and CtBP2+ puncta were processed using the Imaris ‘Spots' module. Classification was based on estimated size, quality and signal intensity. Objects touching the border of the image were excluded. The following build parameters were used for nGFP nuclei: estimated XY diameter=3.50 μm; estimated Z diameter=7.00 μm; ‘Quality' above 20.0; ‘distance to image border XYZ' above 0.001 μm; ‘intensity centre Ch=1' above 1,500. For CtBP2 punta: estimated XY diameter=2.00 μm; estimated Z diameter=5.00 μm; ‘Quality' above 70.0; ‘distance to image border XYZ' above 0.001 μm; ‘intensity centre Ch=2' above 1,500. Of note, these parameters excluded CtBP2+ nuclei and lower intensity CtBP2+ puncta. XY position analysis was performed using the Imaris ‘Vantage' module.

The following antibodies were used: anti-Sox2 (mouse 1:100, BD Biosciences, 561469); anti-myosinVIIa (rabbit 1:100, Proteus, 25–6790); anti-acetylated-α-Tubulin (mouse 1:100, Sigma, T6793); anti-TuJ1 (mouse 1:500, Covance, MMS-435P); anti-Calretinin (Calb2; mouse 1:100, Millipore, MAB1568); anti-CtBP2 (mouse 1:50, BD Biosciences, 612044); anti-Annexin A4 (mouse 1:50, R&D Systems, AF4146); Espin (rabbit 1:100, a generous gift from James Bartles). For most antibodies, mouse embryonic tissue sections were used as positive controls. Mouse embryos were dissected from timed pregnant ICR mice using a protocol approved by the Institutional Animal Care and Use Committee at Indiana University School of Medicine. The embryo fixation and processing procedure was identical to that used for cell aggregates. All secondary antibodies were check for non-specific labelling in the absence of primary antibodies.

### Electrophysiology

On D21 or D22, organoids were shipped overnight from the Hashino Lab in Indianapolis to the Holt Lab in Boston with a small ice pack in Hibernate A medium supplemented with B27, Glutamine, and Normocin (InvivoGen). Upon arrival, they were returned to N2 medium and maintained in culture for up to 6 days, with a partial medium change every other day. Vesicles were dissected from the aggregate and opened with fine dissection scissors, forceps and sharp tungsten needles (Fine Science Tools). For *Atoh1/nGFP* tissue, the organoids were first visualized on an Axiovert 25 inverted microscope equipped with epifluorescence to identify vesicles containing hair cell epithelia and to determine orientation of the patch within the vesicle. After flattening, organoids were stabilized under nylon strands on a glass coverslip.

For utricle hair cells recordings, utricles were excised from P4 (P0 date of birth) Swiss Webster mice, or P3 and cultured overnight in DMEM (Invitrogen) supplemented with 10 mM HEPES, 0.05 mg ml^−1^ ampicillin, and 10 mg l^−1^ Ciprofloxacin (NaOH to pH 7.4) in 5% CO_2_ at 37 °C. The otoconial gel was removed after 5 min of treatment with 0.1 mg ml^−1^ of protease XXIV at room temperature. Hair cells were visualized using DIC microscopy on a Zeiss Axioskop FS with a 63 × water immersion lens. nGFP fluorescence was detected by a filter set with a 495 long-pass filter. To isolate FM4–64 fluorescence, we used the filter set: BP 545/25; FT 570; BP 605/70.

Epithelia were bathed and recorded in artificial perilymph solution containing (in mM): 137 NaCl, 5.8 KCl, 0.7 NaH_2_PO_4_, 10 HEPES, 1.3 CaCl_2_, 0.9 MgCl_2_, 5.6 Glucose, vitamins and essential amino acids (Invitrogen, Carlsbad, CA, USA), adjusted to pH 7.4 with NaOH, ∼310 mmol kg^−1^. Recording pipettes (4–5 mΩ) were pulled from R6 capillary glass (King Precision Glass) and filled with intracellular solution containing (in mM): 135 KCl, 5 HEPES, 5 ethylene glycol tetraacetic acid, 2.5 MgCl_2_, 2.5 K_2_-ATP, 0.1 CaCl_2_, adjusted with KOH to pH 7.4, ∼285 mmol kg^−1^. In some cases we used an internal solution intended to reduce current through K^+^ channels to better isolate other currents; this internal contained: 137 CsCl, 5 EGTA, 5 HEPES, 2.5 Na_2_-ATP, 0.1 CaCl_2_, 3.5 MgCl_2_, pH adjusted with CsOH, ∼290 mmol kg^−1^. A few cells were recorded with 137 TEA-Cl replacing CsCl in the preceding internal solution, to further block K^+^ channels for Ca^2+^ current isolation. Recordings were obtained at room temperature with an Axopatch 200B amplifier (Molecular Devices, Sunnyvale, CA, USA); signals were low-pass filtered at 5 or 10 kHz (Bessel filter) and sampled at 20 kHz with a 16-bit acquisition board (Digidata 1322A) and pClamp 8.2 software (Molecular Devices). Series resistance was compensated at 40%. Voltages were corrected for a calculated liquid junction potential of 5 mV (K internal), 5.9 mV (Cs internal), or −2 mV (TEA internal). Cells held at −65 or −75 mV were included in calculations of prevalence of various currents and transduction, but only cells held at −65 mV were averaged for *I*–*V* curves. Hair bundles were stimulated by drawing the kinocilium into a pipette filled with extracellular solution and held in place with gentle suction. Movement of the stimulus pipette was actuated by a piezoelectric bimorph controlled by a piezo driver (ThorLabs, model MDT694) and filtered at 1 kHz.

### Analysis

Statistical significance was determined using a Student's *t*-test for comparison of two groups or a one-way ANOVA (analysis of variance) followed by Tukey's *post hoc* test for multiple comparisons, unless stated otherwise. All data were analysed using Prism 6 or Microsoft Excel software.

Electrophysiology data were analysed with Clampfit and ORIGIN 2015 (OriginLab) and means are presented±s.e.m. In some cases, multiple files were averaged to improve the signal to noise ratio, data were filtered at 0.5 kHz and capacitive transients were removed for clarity. Input resistance was measured in voltage-clamp as the current change for a 5 mV depolarization. The membrane time constant was measured in current-clamp for a hyperpolarizing step of 5 or 10. For K^+^ current analysis, recordings were accepted if *R*_s_ was no greater than 15 MΩ. As *P*_K_/*P*_Cs_ is 1.17, cells recorded in Cs^+^ and K^+^ internals (depending on other currents being examined) were pooled. Not all measurements could be made in every cell, and *n* values indicate sample size for each measure. Adaptation time constants and extent of adaptation were calculated at half-maximal displacement. Because fast adaptation is sensitive to quality of bundle coupling; cells with poor coupling were excluded from adaptation time course analysis for native and organoid hair cells.

## Additional information

**How to cite this article:** Liu, X.-P. *et al*. Functional development of mechanosensitive hair cells in stem cell-derived organoids parallels native vestibular hair cells. *Nat. Commun.* 7:11508 doi: 10.1038/ncomms11508 (2016).

## Supplementary Material

Supplementary Information Supplementary Figures 1-3

Supplementary Movie 1 Confocal 3D reconstruction and volume rendering of a day 24 ESC-derived organoid containing nGFP+ hair cells. The sensory epithelium is positioned near a neuronal mass containing TuJ1+ neurons (red). Neurites from one or multiple neurons extend into the epithelium. CtBP2+ puncta (white) indicate the sites of putative ribbon synapses between neurons and hair cells.

Supplementary Movie 2 Confocal 3D reconstruction of a day 25 ESC organoid containing Myo7a+ hair cells (red) and TuJ1+ neurites (green).

## Figures and Tables

**Table 1:** Properties of mechanotransduction and general electrophysiology in hair cells from organoids, utricles and values from the literature.

	**ES cell-derived organoid**	**Utricle P4**	**Utricle (Literature)**
Transduction amplitude D24–27 (pA)	144±18 (13)	136±20 (7)	155±16 (36) (ref. 24)
			164±76 (8) (ref. 16)
Transduction 10–90% range (μm)	1.6±0.1 (12)	1.2±0.1 (7)	1.3±0.4 (8) (ref. 16)
Transduction adaptation *τ*_fast_ (ms)	9.1±1.6 (7)	7.6±2.3 (6)	∼3 (15) (ref. 26)
			5.2±0.7 (ref. 24)
Transduction adaptation τ_slow_ (ms)	83.4±8.7 (7)	62.3±6.2 (6)	∼50 (13) (ref. 26)
			49±37 (8) (ref. 16)
			49±6 (9) (ref. 27)
			45.6±4.5 (41) (ref. 24)
Transduction extent of adaptation (%)	44.5±5.0 (7)	66.3±5.4 (7)	∼65% (17) (ref. 26)
			65±3 (ref. 25)
Resting potential Type II (mV)	−63.2±0.7 (38)	−58.2±0.8 (6)	−64±5.2 (98) (ref. 58)
Resting potential Type I (mV)	−79.3±1.6 (4)	−78.1, −86.0 (2)	−77.2±3.1 (62) (ref. 58)
		P5+2DIC	
Input resistance Type II (MΩ)	1,228.7±119.9 (22)	934±85 (12)	1,390±830 (98) (ref. 17)
Input resistance Type I (MΩ)	49.7±11.2 (5)	57.7±14.8 (4)	55±41 MΩ (28) (ref. 17)
		P5+2DIC through P9	
Capacitance (pF)	6.0±0.2 (136)	4.8±0.2 (11)	5.0±1.3 (16) (ref. 17)
Type II			

Organoid data are pooled for all ages D22–28. Our data are presented as mean±s.e.m. and *n* values are noted in parentheses. S.e.m. or s.d. are indicated for literature values where it could be determined. Transduction comparisons are from extrastriolar Type II hair cells.
