# Supplementary material for: Functional development of mechanosensitive hair cells in stem cell-derived organoids parallels native vestibular hair cells
Source: Nat Commun. 2016 May 24;7:11508. doi: 10.1038/ncomms11508 (PMC4890183; doi:10.1038/ncomms11508)
Supplement: Supplementary Information — Supplementary Figures 1-3 [file ncomms11508-s1.pdf]

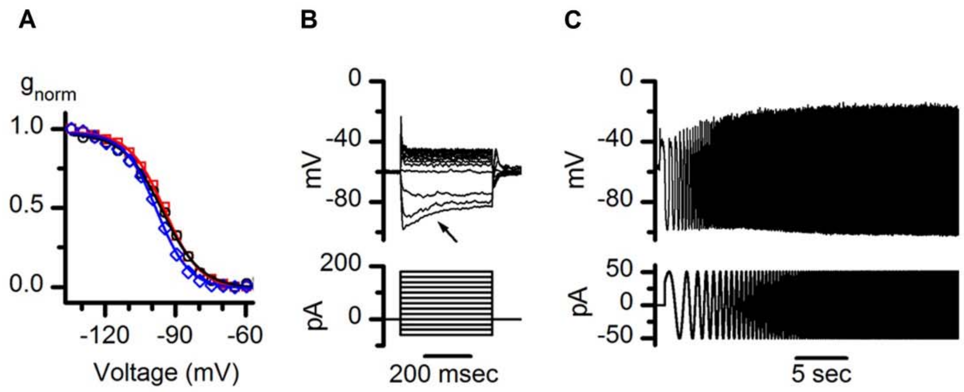

Supplementary Fig. 1. Membrane responses of organoid hair cells. *(A)* Activation curves for I_h obtained from tail currents of three Type II-like organoid hair cells with large I_h. $V_{1/2act}$ and slope values were −95 mV, 7.6; −96 mV, 8.2; and −98 mV, 6.9. *(B)* Family of membrane responses evoked by the protocol shown below. Cells with large I_h showed a depolarization sag (arrow) in response to large hyperpolarizing current steps. *(C)* A continuous frequency sweep from 1 to 30 Hz was injected to examine the membrane frequency response. The organoid cells showed high pass behavior, particularly in the depolarizing direction, up to 30 Hz, a profile typical of vestibular Type II hair cells.

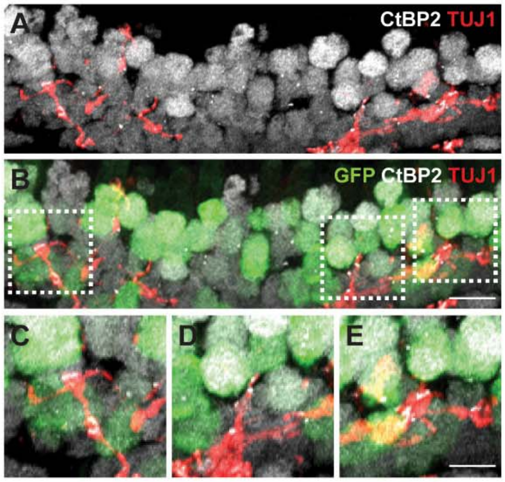

Supplementary Fig. 2. *Atoh1/nGFP* organoid hair cells have features typical of vestibular hair cell synapses. *(A-B)* Confocal image of nGFP+ organoid hair cells with CtBP2+ puncta that colocalize with TUJ1+ neuronal processes. *(C-E)* High magnification images of TUJ1+ processes in contact with CtBP2+ puncta. Scale bar: 10 µm (*B*), 5 µm (E)

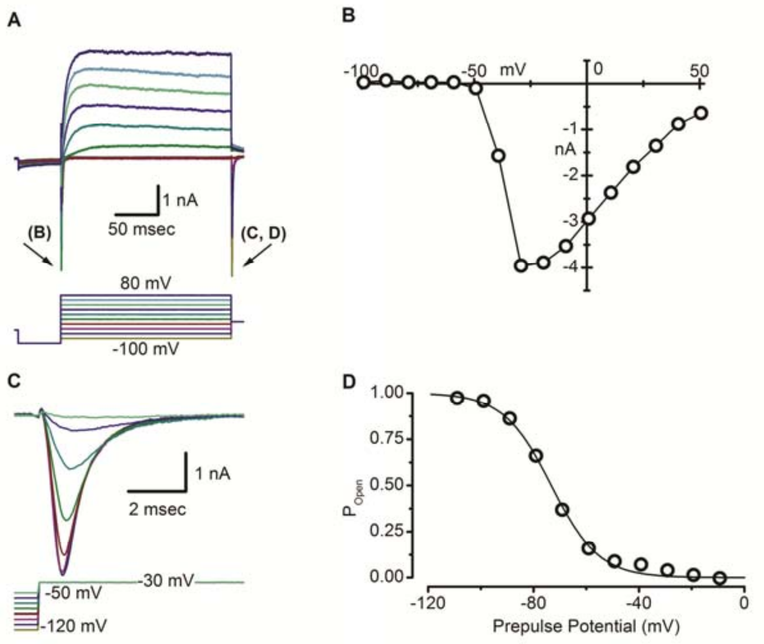

Supplementary Fig. 3. Characterization of voltage-dependent currents recorded from an organoid neuron. (*A*) A family of voltage-dependent currents evoked by the voltage protocol shown below. From the holding potential of −64 mV, the cell was stepped to −120 mV to relieve inactivation and subsequently to potentials between −100 mV and 80 mV in 20 mV increments to activate inward sodium currents and outward potassium currents. (*B*). Peak inward currents, derived from the dataset shown in panel A, plotted as a function of membrane potential resemble those of sodium currents recorded from vestibular ganglion neurons. (*C*) An expanded view of the inward currents, shown in panel A, evoked by steps to −30 mV that were preceded by voltage steps that ranged between −120 and −50 mV. (*D*) Peak inward currents from panel A were normalized and plotted as open probability as a function of prepulse potential. The data were fitted with a first order Boltzmann equation that had a $V_{½}$ of inactivation of −73 mV, *s* of 9 mV Imax of 4.3 nA and Imin of 0.3 nA, consistent with values obtained from sodium current inactivation curves of vestibular ganglion neurons.
